# Impact of Surgeon Experience During Carotid Endarterectomy Operation and Effects on Perioperative Outcomes

**DOI:** 10.5935/1678-9741.20160088

**Published:** 2016

**Authors:** Volkan Yüksel, Ahmet Coskun Ozdemir, Serhat Huseyin, Orkut Guclu, Fatma Nesrin Turan, Suat Canbaz

**Affiliations:** 1 Trakya Üniversitesi Rektörlüğü, Balkan Yerleşkesi, Turkey.

**Keywords:** Endarterectomy, Carotid, Training, Stroke, Treatment Outcome

## Abstract

**Objective:**

We evaluated the effect of surgeon experience on complication and mortality rates of carotid endarterectomy operation.

**Methods:**

Fifty-nine consecutive patients who underwent carotid endarterectomy between January 2013 and February 2016 were divided into two groups. Patients who had been operated by surgeons performing carotid endarterectomy for more than 10 years were allocated to group 1 (experienced surgeons; n=34). Group 2 (younger surgeons; n=25) consisted of patients operated by surgeons independently performing carotid endarterectomy for less than 2 years. Both groups were compared in respect of operative results and postoperative complications.

**Results:**

No intergroup difference was found for laterality of the lesion or concomitant coronary artery disease. In group 1, signs of local nerve damage (n=2; 5.9%) were detected, whereas in group 2 no evidence of local nerve damage was observed. Surgeons in group 1 used local and general anesthesia in 3 (8.8%) and 31 (91.2%) patients, respectively, while surgeons in group 2 preferred to use local and general anesthesia in 1 (4%) and 24 (96%) patients, respectively. Postoperative stroke was observed in group 1 (n=2; 5.9%) and group 2 (n=2; 5.8%).

**Conclusion:**

Younger surgeons perform carotid endarterectomy with similar techniques and have similar results compared to experienced surgeons. Younger surgeons rarely prefer using shunt during carotid endarterectomy. The experience and the skills gained by these surgeons during their training, under the supervision of experienced surgeons, will enable them to perform successful carotid endarterectomy operations independently after completion of their training period.

**Table:** 

**Abbreviations, acronyms & symbols**	
CEA	=Carotid endarterectomy
COPD	=Chronic obstructive pulmonary disease
ICU	=Intensive care unit
PTFE	=Polytetrafluoroethylene
SPSS	=Statistical Package for the Social Sciences
TIA	=Transient ischemic attack

## INTRODUCTION

Carotid artery stenosis leads to stroke and long-lasting disabilities. Atherosclerosis, which settles inside the bifurcation of common carotid artery, is one of the major causes of recurrent ischemic stroke^[^^1^^]^. Current medical approaches aim to slow down the progression of the disease and prevent stroke^[^^2^^]^. Since the first successful carotid endarterectomy (CEA) performed in the 1950s, surgical treatment has become the gold standard in the treatment of carotid stenosis^[^^3^^]^. Its superiority over medical therapy in cases with symptomatic and serious carotid stenosis has definitively been revealed in many studies^[^^4^^-^^6^^]^. CEA is a widely performed procedure in many medical centers, with low complication rates. Within the first 30 postoperative days, local neurological damage, hematoma and bleeding, cardiovascular complications, permanent or transient stroke, and death are the most frequently encountered complications^[^^7^^]^. CEA techniques differ among surgeons; however, no difference regarding postoperative mortality and complications could be demonstrated among those techniques.

In this study, the impact (if any) of surgeon experience on complications and mortality rates of CEA were investigated.

## METHODS

### Study Design

A total of 59 patients (women, n=19; 32%; men, n=40; 68%) who had undergone CEA between January 2013 and February 2016 at our clinic were included in the study. Ethical committee approval for the study was obtained from the local Clinical Research Ethics Committee. Signed informed consent forms were obtained from all patients. All of the study participants consisted of symptomatic patients with a 70-90% carotid stenosis. Medical data and surgical records of the patients were retrospectively examined and the patients were divided into 2 groups. Group 1 consisted of patients (n=34) operated by surgeons who had been performing CEA for more than 10 years (experienced surgeons). The second group (Group 2, n=25) consisted of patients operated by surgeons who had been independently performing CEA for less than 2 years (younger surgeons).

Our clinic has two surgeons who have more than 10 years' experience in CEA and three surgeons who have been performing CEA for less than 2 years. The patients in both groups were compared retrospectively in terms of surgical technique used, postoperative mortality, stroke, bleeding, shunt application, and anesthesia method.

### Surgical Technique

Under local anesthesia, the patients were positioned properly for CEA. The operation site was disinfected with polyvinylpyrrolidone and covered with a sterile drape. To achieve anesthesia, subcutaneous 2% lidocaine was injected. For other patients, general anesthesia was instituted before disinfection and draping steps. A skin incision was made, starting from 2 cm above the sternoclavicular junction up to 2 cm below the earlobe, parallel to the medial edge of the sternocleidomastoid muscle. Subcutaneous tissue and fascial layer were opened, and the common facial vein branch of the internal jugular vein was ligated to access the common carotid artery. Common and internal carotid artery, external carotid artery, and its superior thyroidal branch were suspended with silicon tapes. All patients were heparinized with intravenous 5000 U heparin. Two minutes after heparinization, atraumatic vascular clamps were placed first around the internal, then in the common and external carotid arteries. Afterwards, arteriotomy incision was performed, beginning from the common carotid and extending to the internal carotid artery. Shunt implantation was performed according to surgeons' choice. Endarterectomy was carried out using endarterectomy spatula (Watson-Cheyne dissector) and delicate forceps. Residual intimal tissues on the vessel wall were removed and the lumen was washed using a heparinized isotonic serum. Then, the intimal edges of the common and internal carotid arteries were sutured to the vessel wall with 7/0 propylene sutures. Later, the arteriotomy incision was closed primarily or with a patch. Before ligation of the sutures, clamps and air within the lumen were removed. Then, the suture was ligated and the remaining clamps were removed. Following hemostatic control, a Hemovac drain was placed inside the entry site and fascia, subcutaneous, and cutaneous layers were closed. Intubated patients who received general anesthesia were brought into the intensive care unit (ICU) and connected to an assisted ventilation apparatus. Patients who underwent the procedure under local anesthesia were also transferred to the ICU, and their neurological examinations were performed. Neurological examination of the patients who received general anesthesia was performed after their extubation.

### Statistical Analysis

For statistical analysis, Statistical Package for the Social Sciences 19 (SPSS Inc, Chicago, IL, USA) was used. Compatibility of the measurable data to normal distribution was analyzed using single sample Kolmogorov-Smirnov test and, for intergroup comparisons of those demonstrating normal distribution, independent sample-*t*-test was used. In the evaluation of qualitative data, Fisher's exact test, χ^2^ test with Yates correction, and Kolmogorov-Smirnov two-sample test were used. As descriptive statistics, for measurable data, arithmetic means ± standard deviation, and for quantitative data, numerical values and percentages were provided for all statistical evaluations. *P*<0.05 was considered statistically significant.

## RESULTS

There was no significant intergroup difference between groups in terms of age, gender, cardiovascular disease, smoking, diabetes mellitus, hypertension, hyperlipidemia, chronic obstructive pulmonary disease (COPD), previous peripheral vascular surgery, permanent and transient stroke, and Transient ischemic attack (TIA) (Table 1).

**Table 1:** Patient demographics.

**Variables**	**Group 1 (n=34)**	**Group 2 (n=25)**	***P* value**
Age	65.91±9.16†	67.64±8.95†	0.473*
Gender			
Male	22 (64.7%)	18 (72%)	0.756**
Female	12 (35.3%)	7 (28%)	
Diabetes mellitus	7 (6%)	5 (20%)	1.000**
Hyperlipidemia	10 (29.4%)	8 (32%)	1.000**
Cardiovascular disease	9 (26.5%)	13 (52%)	0.083**
Hypertension	22 (64.7%)	20 (80%)	0.322**
Previous vascular surgery	4 (11.8%)	4 (16%)	0.711***
COPD	3 (8.8%)	1 (4%)	0.630***
CRF	-	1 (4%)	0.424***
Smoking	13 (38.2%)	11 (44%)	0.859**
Previous permanent stroke	9 (26.5%)	6 (24%)	1.000**
TIA	7 (20.6%)	11 (44%)	0.100**

† mean ± standard deviation; COPD=chronic obstructive pulmonary disease; CRF=chronic renal failure; TIA=transient ischemic attack

* Unpaired t test

** Continuity correction test

*** Fisher's exact test

Left and right carotid artery disease were detected in 24 (40%) and 23 patients (38.9%), respectively. Seven patients presented with left carotid artery disease plus coronary artery disease (11.8%) and 5 patients presented right carotid artery plus coronary artery disease (8.4%) (Table 2). No intergroup difference was found for laterality of the lesion or concomitant coronary artery disease (*P*=0.974). In group 1, CEA (n=5, 14.7%), CEA + saphenous vein patch plasty (n=4; 11.8%), CEA +polytetrafluoroethylene (PTFE) graft patch plasty (n=18; 52.9%), combined CEA + PTFE graft patch plasty + coronary bypass (n=5; 14.7%), and combined CEA + saphenous vein patch plasty + coronary bypass (n=2; 5.9%) were performed. In group 2, only CEA (n=2; 8%), CEA + saphenous vein patch plasty (n=2; 8%), CEA + PTFE graft patch plasty (n=15; 60%), CEA + coronary bypass (n=4; 16%), and combined CEA + PTFE patch plasty + coronary bypass (n=1; 4%) were performed. No intergroup difference in terms of surgical technique was observed (*P*=0.852) (Table 3).

**Table 2:** Laterality

**Groups**	**Left carotid disease**	**Right carotid disease**	**Left carotid + coronary artery disease**	**Right carotid + coronary artery disease**	***P* value**
Group 1	12 (35.3%)	16 (47.1%)	4 (11.8%)	2 (5.9%)	0.974*
Group 2	12 (48%)	7 (28%)	3 (12%)	3 (12%)	

* Kolmogorov Smirnov two sample test

**Table 3:** Operative data.

**Variables**	**Group 1 (n=34)**	**Group 2 (n=25)**	***P* value**
x-clamp	33.29±5.09	36.32±4.87	0.025*
Mortality	4 (11.8%)	-	0.130**
Use of shunt	22 (64.7%)	4 (16%)	0.001***
Type of anesthesia			
Local	3 (8.8%)	1 (4%)	0.630**
General	31 (91.2%)	24 (96%)	
Postoperative stroke	2 (6.1%)	2 (7.7%)	1.000**
Nerve damage	2 (5.9%)	-	0.503**

* Unpaired t test

** Continuity correction test

*** Fisher's exact test

In group 1, signs of local nerve damage (n=2; 5.9%) were detected, while in group 2, no evidence of local nerve damage was found. However, no statistically significant difference was observed between the two groups (*P*=0.503). The most important difference between groups is related to the use of shunt implantation. Experienced surgeons performed shunt implantations on 22 (64.7%) patients whereas, in the group of younger surgeons, only 4 (16%) patients had undergone shunt implantation (*P*=0.001). Experienced surgeons used local and general anesthesia in 3 (8.8%) and 31 (91.2%) patients, respectively, while the younger surgeons preferred to use local and general anesthesia in 1 (4%) and 24 (96%) patients, respectively (*P*=0.630). In group 1, 4 (11.8%) patients were lost during the early postoperative period. No cases of mortality were observed in group 2. Nevertheless, there was no statistically significant difference between groups (*P*=0.130). Postoperative stroke was observed in group 1 (n=2; 5.9%) and group 2 (n=2; 5.8%) (*P*=1.000). Three (73%) of those 4 patients were lost in the early postoperative period. Re-exploration because of bleeding was not performed in either group (Table 3).

The surgical procedures varied between the two groups. Table 4 shows the surgical procedures performed in both groups. There was statistically significant difference between groups in terms of procedures performed.

**Table 4:** Procedures.

**Procedures**	**Group 1**	**Group 2**
CEA + primary closure	5 (14.7%)	2 (8%)
CEA + saphenous vein patch plasty	4 (11.8%)	2 (8%)
CEA + PTFE patch plasty	18 (52.9%)	15 (60%)
CEA + CABG	-	4 (16%)
CEA + saphenous vein patch plasty + CABG	2 (5.9%)	1 (4%)
CEA + PTFE patch plasty + CABG	5 (14.7%)	1 (4%)
*P* value	0.852*	

CABG=coronary artery bypass grafting; CEA=carotid endarterectomy; PTFE=polytetrafluoroethylene

* Kolmogorov Smirnov two sample test

## DISCUSSION

Carotid artery stenosis is an important health problem and a significant cause of stroke and mortality. Superiority of CEA in the prevention of stroke in cases with carotid stenosis has been established^[^^8^^]^. Selection of patients for CEA is a very important issue. In determining the treatment modality for carotid artery stenosis, five distinct conditions should be considered^[^^2^^]^: 1. Neurological symptoms; 2. Severity of carotid stenosis; 3. Medical comorbidities; 4. Vascular and local anatomic features: 5. Carotid plaque morphology.

Generally, for invasive intervention, features of items 1 and 2 are considered and, when choosing between surgery and carotid stenting, characteristics of items 3, 4, and 5 are considered.

Many conditions influence the success of CEA. To establish indications for CEA, consideration of the aforementioned conditions, surgical adequacy, and surgical experience play important roles, as is the case for all peripheral vascular interventions. Many published research studies have concluded that surgeons performing fewer number of endarterectomies encountered higher incidences of stroke and death^[^^9^^]^. Many studies have compared trainees performing CEA under supervision to surgeons who carried out CEA independently, and generally those investigations could not detect any difference between surgical procedures applied in terms of stroke and death rates^[^^10^^-^^12^^]^. Different from these studies, in this investigation, experienced surgeons who practiced CEA for more than 10 years and those performing CEA independently for less than 2 years without any supervision were compared. No intergroup difference in terms of stroke and death rates was observed. Stroke and death rates in both groups are compatible with the results reported by the European Carotid Surgery Trial, and North American Symptomatic Carotid Endarterectomy Trial surveys.

Rationale for the preference for either local or general anesthesia between groups is almost the same. In our clinic, we prefer to perform all CEAs under general anesthesia. In cases with contralateral carotid occlusion or advanced carotid stenosis, local anesthesia may confer some benefits^[^^13^^,^^14^^]^. In our study, one patient developed malign hyperthermia secondary to general anesthesia and was lost.

Transient interruption of cerebral blood flow during CEA could be prevented by shunt implantation. However, shunt implantation during CEA is not a routine practice in our clinic, and any evidence that requires application of shunt is lacking^[^^2^^]^. Shunt implantation may result in the risk of embolization and dissection^[^^15^^]^. Generally, the presence of contralateral carotid occlusion or serious stenosis in addition to routines and preferences of the surgeon are determining factors for the application of a shunt. In our study, shunt implantation in group 1, which encompassed experienced surgeons, was found to be relatively more frequent (*P*<0.001). Any intraoperative complication secondary to shunt implantation was not detected.

During CEA, closure of arteriotomy incision using vein or synthetic patch can decrease the rate of arterial restenosis^[^^16^^,^^17^^]^. A patch was used in 42 (71%) of our 59 patients. No intergroup difference as for patch application was observed. Routinely, saphenous vein was used as a venous patch because of higher rates of restenosis with Dacron patches; a synthetic PTFE patch was also employed^[^^18^^,^^19^^]^.Since saphenous vein harvested from the ankle region is more prone to rupturing when compared with a saphenous vein segment resected above the knee, harvesting saphenous vein segment from inguinal or above-the-knee was preferred^[^^18^^]^.

## CONCLUSION

In conclusion, younger surgeons perform CEA operations with similar techniques and have similar results compared to experienced surgeons. Younger surgeons rarely prefer using shunt during CEA operations. When carrying out risky and challenging procedures like CEA, to be under the supervision of experienced surgeons is an important routine that makes trainees feel safe in their applications. The experience and skills gained by these surgeons during their training under the supervision of experienced surgeons will enable them to perform successful and safe CEA operations independently after completion of their training period.

**Table:** 

**Authors' roles & responsibilities**	
VY	Conception and study design; analysis and/or data interpretation; manuscript writing or critical review of its content; final manuscript approval
ACO	Conception and study design; analysis and/or data interpretation; manuscript writing or critical review of its content; final manuscript approval
SH	Conception and study design; realization of operations and/or trials; manuscript writing or critical review of its content; final manuscript approval
OG	Analysis and/or data interpretation; statistical analysis; manuscript writing or critical review of its content; final manuscript approval
FNT	Analysis and/or data interpretation; statistical analysis; final manuscript approval
SC	Conception and study design; manuscript writing or critical review of its content; final manuscript approval

## References

[1] Veith FJ, Amor M, Ohki T, Beebe HG, Bell PR, Bolia A (2001). Current status of carotid bifurcation angioplasty and stenting
based on a consensus of opinion leaders. J Vasc Surg.

[2] Liapis CD, Bell PR, Mikhailidis D, Sivenius J, Nicolaides A, Fernandes e Fernandes J, ESVS Guidelines Collaborators (2009). ESVS guidelines. Invasive treatment for carotid stenosis:
indications, techniques. Eur J Vasc Endovasc Surg.

[3] Duncan JM, Reul GJ, Ott DA, Kincade RC, Davis JW (2008). Outcomes and risk factors in 1,609 carotid
endarterectomies. Tex Heart Inst J.

[4] Luebke T, Aleksic M, Brunkwall J (2007). Meta-analysis of randomized trials comparing carotid
endarterectomy and endovascular treatment. Eur J Vasc Endovasc Surg.

[5] Halliday A, Mansfield A, Marro J, Peto C, Peto R, Potter J, MRC Asymptomatic Carotid Surgery Trial (ACST) Collaborative
Group (2004). Prevention of disabling and fatal strokes by successful carotid
endarterectomy in patients without recent neurological symptoms: randomised
controlled trial. Lancet.

[6] Hobson RW, Weiss DG, Fields WS, Goldstone J, Moore WS, Towne JB (1993). Efficacy of carotid endarterectomy for asymptomatic carotid
stenosis. The Veterans Affairs Cooperative Study Group. N Engl J Med.

[7] Kragsterman B, Logason K, Ahari A, Troëng T, Parsson H, Bergqvist D (2004). Risk factors for complications after carotid endarterectomy: a
populationbased study. Eur J Vasc Endovasc Surg.

[8] Organ N, Walker PJ, Jenkins J, Foster W, Jenkins J (2008). 15 year experience of carotid endarterectomy at the Royal
Brisbane and Women's Hospital: outcomes and changing trends in
management. Eur J Vasc Endovasc Surg.

[9] Ruby ST, Robinson D, Lynch JT, Mark H (1996). Outcome analysis of carotid endarterectomy in Connecticut: the
impact of volume and specialty. Ann Vasc Surg.

[10] Rijbroek A, Wisselink W, Rauwerda JA (2003). The impact of training in unselected patients on mortality and
morbidity in carotid endarterectomy in a vascular training center and the
recommendations of the European Board of Surgery Qualification in Vascular
Surgery. Eur J Vasc Endovasc Surg.

[11] Lutz HJ, Michael R, Gahl B, Savolainen H (2009). Is carotid endarterectomy a trainee operation. World J Surg.

[12] Pai M, Handa A, Hands L (2002). Adequate vascular training opportunities can be provided without
compromising patient care. Eur J Vasc Endovasc Surg.

[13] Lewis SC, Warlow CP, Bodenham AR, Colam B, Rothwell PM, Torgerson D, GALA Trial Collaborative Group (2008). General anaesthesia versus local anaesthesia for carotid surgery
(GALA): a multicentre, randomised controlled trial. Lancet.

[14] Mendonça CT, Fortunato JA, Carvalho CA, Weingartner J, Filho OR, Rezende FF (2014). Carotid endarterectomy in awake patients: safety, tolerability
and results. Rev Bras Cir Cardiovasc.

[15] Whiten C, Gunning P (2009). Carotid endarterectomy: intraoperative monitoring of cerebral
perfusion. Curr Anaesth Crit Care.

[16] Katz D, Snyder SO, Gandhi RH, Wheeler JR, Gregory RT, Gayle RG (1994). Longterm follow-up for recurrent stenosis: a prospective
randomized study of expanded polytetrafluoroethylene patch angioplasty
*versus* primary closure after carotid
endarterectomy. J Vasc Surg.

[17] AbuRahma AF, Khan JH, Robinson PA, Saiedy S, Short YS, Boland JP (1996). Prospective randomized trial of carotid endarterectomy with
primary closure and patch angioplasty with saphenous vein, jugular vein, and
polytetrafluoroethylene: perioperative (30 day) results. J Vasc Surg.

[18] Naylor R, Hayes PD, Payne DA, Allroggen H, Steel S, Thompson MM (2004). Randomized trial of vein versus dacron patching during carotid
endarterectomy: long-term results. J Vasc Surg.

[19] AbuRahma AF, Hannay RS, Khan JH, Robinson PA, Hudson JK, Davis EA (2002). Prospective randomized study of carotid endarterectomy with
polytetrafluoroethylene versus collagen impregnated Dacron (Hemashield)
patching: perioperative (30-day) results. J Vasc Surg.

